# Geology, Introductory, Descriptive, and Practical

**Published:** 1845-04

**Authors:** 


### Art. XIX.

*Geology, Introductory, Descriptive, and Practical.* By David Thomas Ansted, m.a. f.r.s., Professor of Geology in King's College, London. In Two Volumes.—*London*, 1844. 8vo, pp. 1078.

Few of our readers, we should hope, are unacquainted with the beautiful series of illustrated works on various departments of natural history, for which the public is indebted to the enterprise of Mr. Van Voorst, and the co-operation of the able authors who have contributed to it; and to say that the present volumes are fully entitled to take rank among them, is no mean praise. Although but a young man, Professor Ansted has long been known for the zeal and ability with which he has prosecuted various departments of geological science, under the guidance of the veteran Sedgwick; and his work may be regarded as based on the general principles held by the Cambridge professor, as well as an exposition of his opinions upon many topics at present under discussion. Our own judgment of it has been formed upon a careful perusal of nearly the whole treatise; and knowing that it coincides with that of many distinguished geologists, we do not hesitate to express the belief that it is better adapted to convey to the students a competent knowledge of the *certainties* of the science, than any other with which we are acquainted. To all who are interested in the practical operations to which geology is subservient, and which are now so extensively aided by its means, Professor Ansted's work will be peculiarly welcome, on account of the large quantity of information which it contains on these subjects. Those who seek an array of captivating theories or ingenious hypotheses, must seek elsewhere, since it does not fall within the author's plan to discuss them. The following extracts from his introduction will give a fair idea of the plan of his work:

"In the work I am now undertaking, it is my object to acquaint the reader with the nature of the *foundations* of the science of geology; and the superstructure—by which I understand the theories and the laws deduced—will not be allowed to detain us long, because, however interesting they may be, they can only be instructive to the advanced and perfect geologist. I trust, however, I shall be able to show that the facts themselves upon which the theories are founded, are sufficiently numerous and important to deserve the most careful consideration; and that the acquirement of a general knowledge of these facts is, therefore, an object not unworthy the serious attention of all who are interested in the works of nature; and that it is an object not only deeply interesting, but permanently and immediately useful, and eminently practical; and lastly, that such knowledge is to be acquired—as indeed useful knowledge of all kinds must be acquired—not by wildly and weakly systematizing, and making gratuitous suppositions to strengthen groundless hypotheses, but by calm, sober observation, founded upon and directed by an acquaintance with what has been already effected, and having constantly in view the elucidation of truth, not the establishment of a theory." (p. 3.)

When we add that the work is embellished with a profusion of woodcut illustrations, some of them geological diagrams, whilst others are delineations of the most important fossil remains of plants and animals—all executed with the utmost care and in the very best style of the art, we think we have said enough to recommend it to the attention of our readers.

